# Flexible and rapid validation of structural variation using adaptive sampling

**DOI:** 10.1038/s41431-026-02039-4

**Published:** 2026-02-23

**Authors:** Aida Paivandy, Felix Lenner, Jesper Eisfeldt, Tord Jonson, Hans Ehrencrona, Anna Lindstrand, Stephen W. Scherer, Lars Feuk

**Affiliations:** 1 Department of Immunology, Genetics and Pathology, Uppsala University, Uppsala, Sweden; 2 SciLifeLab, Uppsala University, Uppsala, Sweden; 3 Department of Molecular Medicine and Surgery, Karolinska Institutet, and Department of Clinical Genetics and Genomics, Karolinska University Hospital, Stockholm, Sweden; 4 Division of Clinical Genetics, Department of Laboratory Medicine, Lund University, Lund, Sweden; 5 Department of Clinical Genetics, Pathology and Molecular Diagnostics, Skåne University Hospital, Lund, Sweden; 6 The Centre for Applied Genomics and Program in Genetics and Genome Biology, The Hospital for Sick Children, Toronto, Canada; 7 McLaughlin Centre and Department of Molecular Genetics, University of Toronto, Toronto, Canada

**Keywords:** Genetics research, Translational research

## Abstract

Identification of genomic rearrangements by microarrays or short-read sequencing frequently lacks information about the exact architecture and breakpoints of variants due to technical limitations. Independent verification of complex structural variants (SVs) is often performed using custom targeted assays, making confirmation of clinically relevant findings time consuming and laborious. In this study we evaluate Oxford Nanopore long-read adaptive sampling for flexible and rapid confirmation and characterization of complex genomic rearrangements and structural variants. Adaptive sampling is an in silico target enrichment, where continued sequencing or ejection of a fragment is based on whether it matches a defined reference sequence. Using adaptive sampling, we targeted 10 regions with different structural variant types, including deletions, translocations, and complex rearrangements. Each sample was analyzed on a MinION or PromethION flow-cell, and sequencing resulted in between 14.1–18.3 Gb of data per sample, with mean autosomal on-target coverage of 28.4x and off-target read depth coverage of 5.3x. We were able to verify all 10 rearrangements, with breakpoint spanning reads for nine of the ten regions, and fully resolved the architecture of nine regions. We also show that background reads can be used to detect structural variants in non-targeted regions of the genome. Our results show that adaptive sampling represents a flexible and rapid strategy for confirmation and characterization of clinically relevant genomic rearrangements in clinical samples. By providing sequence information, read depth, and methylation data, nanopore adaptive sampling has advantages over other assays for variant confirmation used in diagnostic laboratories today.

## Introduction

Structural variants (SVs) play a crucial role in shaping genetic diversity, contributing to both normal variation and pathological conditions [1]. SVs are generally defined as variants >50 base-pairs (bp) in size, and every genome carries approximately 20–25 thousand structural variants [2], primarily in the form of insertions and deletions [3]. The majority of SVs in a given genome are <1 kilobase (kb) in size and have no measurable phenotype association. In contrast, large (>100 kb) and rare SVs are more likely to cause genomic perturbations and lead to phenotypic consequences [4]. SVs >1 kb in size are often referred to as copy-number variants (CNVs) if they are gains or losses [1], while larger variants involving repositioning of genetic material are commonly called chromosomal rearrangements. In the genetic diagnostic clinic, screening for large, rare, or de novo SVs is typically performed in patients with neurodevelopmental disorders, congenital malformation syndromes, or cancers, since SVs have been shown to be a significant contributor to pathogenesis in these patient groups [5].

Over time, there has been a transformative evolution in the methods used to analyze SVs. In genetic diagnostics, cytogenetic methods and later chromosomal microarray analysis (CMA) have long been used to screen for chromosomal aberrations. More recently, short-read sequencing of either exomes or whole genomes is gradually replacing CMAs as a first-line diagnostic test [6, 7]. However, both CMAs and short-read sequencing approaches have limitations. CMA can only detect CNVs, and has limited resolution in breakpoint identification. Exome sequencing suffers from similar limitations in terms of SV analysis. Short-read whole genome sequencing (WGS) has the potential to detect all different types of SVs, but detection is sequence context dependent, and it has become clear that only a fraction of all SVs in a genome are identified using short-read sequencing [8]. In addition, characterization of the architecture of chromosomal rearrangements is challenging, and breakpoints can often not be identified at nucleotide resolution.

A specific challenge with complex SVs in the diagnostic setting is the verification of variants following initial identification with CMA, exome sequencing, or WGS. Complex chromosomal rearrangements can often be identified but not fully resolved [4]. Validation using FISH or other probe-based quantification methods, such as Multiplex ligation-dependent probe amplification (MLPA) or quantitative PCR (qPCR) can be used for verification of presumed structures, but does not provide further information about SV architecture or breakpoints. In addition, for rare or unique events, these validation assays require new probes or primers to be ordered, followed by experimental optimization, causing delays of several days to weeks before confirmation of the structural variant can be performed.

The advent of long-read sequencing technologies has addressed many of the challenges encountered by CMA and short-read analysis by offering extended reads, allowing researchers to navigate through repetitive regions and identify intricate structural rearrangements with significantly enhanced precision. This shift in sequencing methodologies not only enhances our ability to identify and categorize SVs but also holds promise in revealing new aspects of the genetic landscape linked to biological outcomes. Using long-read sequencing, SVs are more easily resolved, and breakpoint junctions can more reliably be identified at nucleotide resolution, even within repetitive regions. Identification of exact breakpoint sequences helps explain the mechanism of variant formation and may be important for accurate counseling with regard to recurrence risk, optimal and personalized treatment. Targeted long-read sequencing across rearrangement regions would also be a way to verify complex variants, while also providing additional data, including exact breakpoint information, resolution of rearrangement architecture, and enabling phasing of variation regions.

Targeted long-read sequencing can be performed using both PacBio and Oxford Nanopore Technologies (ONT) sequencing. While long-range PCR can be used for target enrichment, it is not ideal as it may introduce PCR bias and make it challenging to cover large regions. Protocols to perform long-read sequencing of native DNA are also available for both PacBio and ONT sequencing. One strategy is to use CRISPR-Cas to excise the region of interest [9, 10]. There are many benefits to use native DNA in combination with long-read sequencing, as it enables haplotype phasing and provides DNA modification information [11, 12]. However, the CRISPR-Cas protocols still require unique probes to be designed and ordered for each target region. More recently, an in silico targeting strategy called adaptive sampling was introduced, which works uniquely for ONT sequencing, building on the rapid base-calling and control of each individual pore on the nanopore flow cell [13]. Adaptive sampling provides a computational approach for target enrichment by comparing fragments being sequenced by each pore to a defined reference, and rejecting those reads not matching the reference file [14, 15]. There is a range of applications where adaptive sampling has the potential to replace experimental targeting workflows and provide a more rapid diagnostic test. A number of studies have shown the potential of adaptive sampling diagnostics in pharmacogenomics [16], cancer [17], repeat disorders [18], and the identification of SVs in rare disease [19].

In this study, we implemented adaptive sampling as a strategy for flexible, rapid validation of chromosomal rearrangements. Targeted ONT sequencing was used to verify and resolve clinically relevant structural variations and complex rearrangements in patients with neurodevelopmental disorders. We discuss the benefits and limitations of the approach, and by targeting variants of different types, sizes, and complexities, we show that nanopore sequencing with adaptive sampling provides an excellent strategy for independent verification of SVs and chromosomal rearrangements identified by clinical microarrays or whole genome sequencing.

## Methods

### Sample selection

Individuals included in the study carry large structural genetic variants that have been previously characterized by short-read or long-read whole-genome sequencing. A subset of the samples was selected from the autism spectrum disorder project MSSNG [20], while the remaining individuals were from Swedish clinical genetics units (sample information is summarized in Supplementary Table 1). The samples were included in previous whole-genome sequencing studies [21–24].

### DNA extraction, quality assessment, and processing

Genomic DNA was extracted either from whole blood or immortalized lymphoblastoid cell lines. DNA from cell pellets was extracted with the Nanobind CBB Big DNA Kit (Part Number NB-900-001-01) according to the HMW protocol for Cultured Mammalian Cells in the Nanobind CBB Big DNA Kit Handbook v1.8. DNA from whole blood was extracted using Qiasymphony according to standard protocols (Qiagen, Venlo, Netherlands). The quality of DNA (A_260_/A_280_ and A_260_/A_230_ ratios) was assessed using a NanoDrop 2000 spectrophotometer (Thermo Fisher Scientific, Waltham, Massachusetts, USA), and the concentration was determined using a Qubit fluorometer dsDNA BR Assay Kit (Q33265, Thermo Fisher Scientific) and Qubit 4 Fluorometer (Thermo Fisher Scientific). The length distribution of the DNA was evaluated using a Femto Pulse system (Agilent Technologies, Santa Clara, California, USA). If the samples were viscous and the Femto Pulse system measurement revealed a distinctive peak corresponding to very large DNA fragments (larger than 100 Kbp), a Megaruptor shearing was employed using Megaruptor® 3 (Diagenode, Liege, Belgium), to target a size of approximately ~70 kbp (concentration of 50 ng/µl, 150 μl volume, speed = 001). For samples where fragments smaller than 10 kbp in the fragment-length distribution graph of Femto Pulse exceeded 10%, short read elimination was applied using Short Read Eliminator; SRE XS kit (#102-208-200, Pacific BioSciences, California, USA). Next, DNA fragments were sheared using Covaris g-TUBEs (#520079, Covaris) with two passages at 4400 r.p.m for 4 min centrifugation (#5415 R, Eppendorf). According to the manufacturer's protocol, this shearing should be used for a target size of approximately 20 kbp, but in our experience, these settings result in fragment sizes in the range of 8–10 kb. After shearing, the DNA was end-repaired by subjecting it to the NEBNext® companion module reagents (E7180, New England Biolabs), according to the manufacturer’s recommendations.

### Library preparation and sequencing

The library was prepared with 1.5-2 µg DNA as starting material using the Oxford Nanopore Technologies (ONT) ligation sequencing kit (SQK-LSK114, ONT) together with Sequencing Auxiliary Vials (EXP-AUX003) following the manufacturer’s instructions for MinION. Multiplexed library preparation for P2 Solo was performed using the native barcoding kit 24 V14 (SQK-NBD114.24) together with the native barcoding auxiliary kit V14 (EXP-NBA114). Approximately 20-30 fmol library was loaded onto a MinION flow cell (R10.4.1) and 40-50 fmol onto a PromethION flow cell (R10.4.1). MinION sequencing was run for up to 72 hours on a MinION Mk1B with MinKNOW version 23.04.5. Additionally, a P2 Solo (P2S) device with MinKNOW version 23.07.12 was used to run one barcoded PromethION flow cell. To obtain sufficient data and increase the yield, flow cells were washed (EXP-WSH004, ONT), incubated for 1-2 h, and reloaded one or two times after approximately 24 h and 48 h, respectively.

### Adaptive sampling

MinION sequencing was run with the device connected to a Legion T7, Core i7, 32 GB RAM computer with an RTX3080 graphics card. For MinION sequencing, adaptive sampling was configured to run in enrich mode and the Fast model, using a custom fasta-file containing the regions of interest. DNA sequences representing regions of interest were cut out from the Genome Reference Consortium Human Build 38 (GRCh38), with the exception of one region with a gap in the GRCh38 reference. In the latter case, the region of interest was extracted from the T2T-CHM13v2.0 (T2T-CHM13) assembly [25]. For the multiplexed P2 Solo sequencing, a BED-file containing the regions of interest was supplied together with the GRCh38 reference, excluding the T2T-CHM13 region. This was due to using an updated MinKNOW version (23.07.12) during the P2 Solo sequencing, where using a fasta-only strategy was likely to result in low enrichment. In all samples, seven regions (A, B, C, D, E, F, G) - including both control and rearrangement regions - ranging between 100 kb and 9 Mb were targeted. After sequencing the first three samples using these regions, new targets were added incrementally as additional samples were sequenced (Supplementary Table 1). The maximum amount of targeted sequence in a run amounted to approximately 36 Mb, or 1.2% of the genome.

### Data analysis

Raw sequencing data were re-basecalled (and demultiplexed in the P2 Solo run) with Dorado version 0.5.1 (https://github.com/nanoporetech/dorado) using the super-accurate model (dna_r10.4.1_e8.2_400bps_sup@v4.3.0), while calling methylated bases with the accompanying v1 version of the ‘5mCG_5mhCG’ modified-base model. In runs where all or some of the sequencing data had been saved as fast5 files, these were converted to pod5 files using Pod5 version 0.3.2 (https://github.com/nanoporetech/pod5-file-format) before re-basecalling. Reads with an average quality score below 10 were then discarded using SAMtools 1.17 [26], before mapping the reads to GRCh38 and T2T-CHM13 using minimap2 [27]. Structural variants were called using Sniffles2 version 2.0.7 [28] with default parameters, and complemented by manual inspection in IGV. The read depth across target regions was extracted from the aligned BAM-files using mosdepth [29], while supplying the target regions as a BED-file.

On-target reads were defined as reads “accepted” by adaptive sampling (“end_reason” not equal to “data_service_unblock_mux_change” in the sequencing summary sheets from MinKNOW), while off-target reads were defined as rejected reads where “end_reason” was equal to “data_service_unblock_mux_change”.

CNV analysis using read depth across target regions was calculated in 500 bp bins using mosdepth. All reads passing quality filters that aligned within the target-regions were used. The mean depth in smaller windows across the target regions was then compared to the mean coverage of all target regions in each sample (chromosome X regions excluded in male samples), using a log2 ratio cut-off as described in Greer et al. [19]. For background CNV analysis, only off-target reads as determined by adaptive sampling were used, since a small number of “off-target” reads will later align to the target regions, and a small number of “on-target” reads will align outside the target regions, creating regions with (falsely) high coverage. Depth was first extracted in 10 kb bins across the genome using mosdepth, then the mean depth across 1 Mb regions was compared to the mean coverage of each chromosome. Common CNV regions and regions with unreliable and variable coverage (e.g., centromeres and telomeres) were excluded: (https://github.com/PacificBiosciences/HiFiCNV/tree/7b0622788cbfbf571c34fff55924991b6c688893/data/excluded_regions).

## Results

**Fig. 1:**
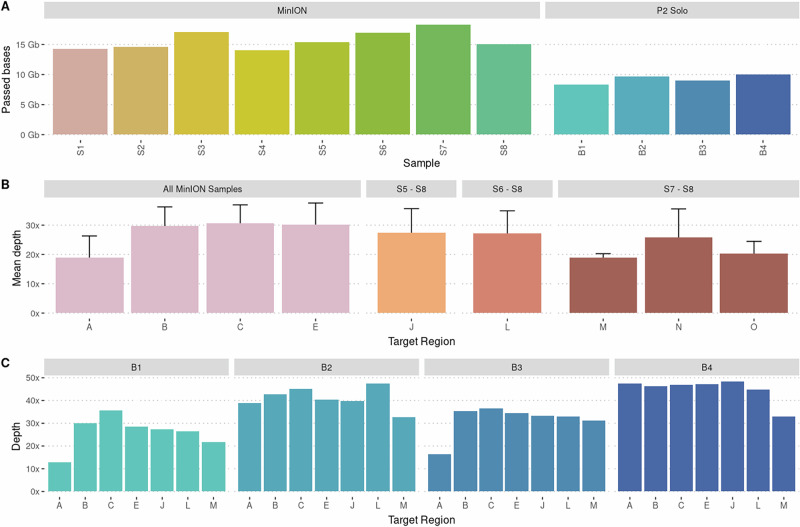
Overview of sequencing results. **A** Bases that passed quality filters for each sample run as single samples on MinION flowcells. **B** Depth of target regions from GRCh38 for the samples sequenced on MinION. Regions A–E included in all samples, J in samples S5 - S8, L in samples S6–S8, and M, N, and O in samples S7 and S8. **C** Target region depth across four multiplexed samples (B1–B4) on a PromethION flow cell. The same regions were targeted in all four samples.

In total, we targeted 10 different chromosomal regions using adaptive sampling and MinION nanopore sequencing (Table 1), and also evaluated the use of the P2 sequencer for a subset of samples. Targets were selected to include different types of rearrangements, ranging from variants with low complexity (deletions, translocations) to complex rearrangements with multiple breakpoints. MinION sequencing was performed with one sample per flow cell (8 samples in total). After quality filters, the sequencing resulted in between 14.1 – 18.3 Gb of data (Fig. 1A). The mean on-target read N50 was 11.9 kb (ranging from 9.6 to 16.0 kb per sample), while the mean off-target read N50 was 569 bp (546 - 595 bp). The mean autosomal depth across targets aligned to GRCh38 (region K from CHM13-T2T excluded) was 28.4x (ranging from 17.4x to 41.6x), while off-target mean coverage was 5.3x. The overall genome coverage ranged between 4.5 and 6.2x, resulting in a target enrichment of 3.2-7.4x (mean 5.3x). Statistics summarizing the results is shown in Table 2. Although the number of target regions varied between samples (Supplementary Table 1), there was no significant correlation between target size and read coverage.

**Table 1:** List of targeted regions. Coordinates from GRCh38 if not otherwise stated.

Target Region	Coordinates	Variant type	Target Size	Platform
A	chrX:27,000,000-36,000,000	Complex (Dup-Nml-Dup)	9 Mb	MinION+P2S
B	chr14:20,000,000-22,500,000	Complex (Dup-Nml-Dup)	2.5 Mb	MinION+P2S
C	chr16:2,500,000-5,000,000	Complex (Trip-Quint-Trip)	2.5 Mb	MinION+P2S
E	chr4:108,000,000-115,000,000	Complex (Dup-Trip-Dup)	7 Mb	MinION
J	chr6:96,600,000-97,300,000	Deletion	700 Kb	MinION
K	chr6:95,868,323-96,766,693*	Translocation	898 kb	MinION
L	chr17:4,825,565-4,925,565	Translocation	100 Kb	MinION
M	chr4:181,364,917-181,464,917	Translocation	100 Kb	MinION+P2S
N	chr9:12,993,905-13,093,905	Translocation	100 Kb	MinION+P2S
O	chr16:28,242,001-29,708,000	Deletion	1.47 Mb	MinION

*Coordinates in T2T-CHM13 assembly. Dup – duplication, Nml – normal copy number, Trip – triplication, Quint – quintuplication, P2S – PromethION 2 Solo.

**Table 2:** Overview of sequencing statistics from the MinION and P2 Solo sequencing experiments.

Platform	Off-target read N50	On-target read N50	Autosomal on-target coverage	Chromosome X coverage	
				Male	Female
MinION	569 bp (546 - 595 bp)	11.9 kb (9.6 - 16.0 kb)	28.4x (17.4 - 41.6x)	14.7x (9.9x - 23.6x)	26.7x (22.2x - 32.4x)
P2S	526 bp (521 - 521 bp)	10.4 kb (9.5 - 11.5 kb)	37.0x (21.8x – 54.2x)	15.2x (13.0x - 17.2x)	42.7x (37.1 - 47.9x)

Autosomal and chromosome X coverage reported as mean values per sample and region with minimum and maximum reported coverage in parenthesis.

To evaluate the potential for multiplexing, four barcoded samples were also run together on one PromethION flow cell on the P2 Solo device. The P2 Solo experiment generated 8.4–10.1 Gb of bases per barcode passing quality filters (Fig. 1A, Supplementary Table 1). On-target reads had a mean N50 read length of 10.4 kb (ranging from 9.5–11.5 kb per sample), while off-target reads had a mean N50 of 526 bp (521–538 bp). The mean autosomal depth across target regions was 37.0x, ranging from 21.8x to 54.2x. The overall genome coverage ranged between 2.9 and 4.6x per barcode (mean 3.8x), resulting in an enrichment between 7.2 and 12.3x (mean 9.8x).

### Characterization of structural variants

All samples have previously been analyzed by whole-genome sequencing, with breakpoints defined either through long-read approaches [21, 22] (PacBio or Oxford Nanopore Technologies) or to a larger region by fluorescence in situ hybridization (target Region K) [24]. Targets were selected to range from simple rearrangements with single breakpoints (translocations, deletions) to complex variants with multiple breakpoint junctions. All 10 rearrangements that were targeted could be verified through adaptive sampling, using either read depth, identification of breakpoint spanning reads, or a combination of both (Supplementary Figs. 1–8). The result for each region is described in Supplementary Table 1. The combination of read depth and breakpoint information enabled full resolution of rearrangement architecture, with the exception of Region C, where three different solutions are possible despite identification of each breakpoint (as previously described in [22]). For this region, reads spanning across a > 400 kb block would be required to fully resolve the structure. Breakpoint spanning reads were identified for all regions (Supplementary Figs. 1–8) except for a 16p11.2 deletion (Region O), where the breakpoints are located in long segmental duplications and the specific nucleotide resolution breakpoint could not be unambiguously determined.

**Fig. 2:**
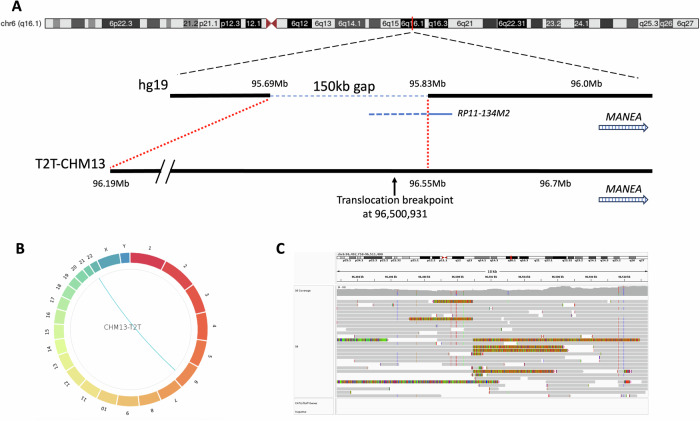
Identification of a translocation on chromosome 6, by using sequence from the CHM13-T2T assembly. **A** Overview of the breakpoint region in hg19 and T2T-CHM13 assemblies. Nearby gene *MANEA* is shown for reference. The breakpoint was previously mapped by FISH to a gap in the hg19 assembly using a BAC probe (RP11-134M2) anchored on the telomeric side of the gap region. The gap region, estimated to 150 kb in hg19, corresponds to a > 360 kb region in T2T-CHM13. Targeting this sequence with adaptive sampling led to the identification of the exact chr6 breakpoint. **B** Circos plot showing the (t6;22)(q16.1;p13) translocation. **C** IGV image showing reads spanning the translocation breakpoints, which can be mapped at nucleotide resolution on chr6, while the breakpoint on the short arm of chr22 maps to a region of identical repeats where no exact breakpoint could be established.

One individual (sample S6) carried a chr6 deletion with a nearby translocation (t6;22)(q16.1;p13) that had previously been characterized using fluorescence in situ hybridization (FISH) and short-read whole-genome sequencing [24]. However, the breakpoint had only been mapped using a bacterial artificial chromosome (BAC) FISH probe, and the exact breakpoint had not been possible to identify at nucleotide resolution. Based on prior analysis, the breakpoint was concluded to be within chr6:95.5–96.8 Mb (hg19), which contains assembly gaps in the GRCh38 reference. Therefore, the corresponding region from T2T-CHM13 (T2T) genome assembly was used as input for adaptive sampling. Using the T2T assembly, the exact translocation breakpoint on chromosome 6 was identified at chr6:96,500,931 (Supplementary fig. S6e). The chr22 breakpoint mapped to the heterochromatic short arm in sequence present in T2T (approximate breakpoint at chr22:3,117,669), which is missing in hg19 and hg38 (Fig. 2). The downstream deletion in the same patient was also confirmed, with start and end positions matching previous short-read findings (Supplementary fig. S6a–d).

### Verification of copy number variants

**Fig. 3:**
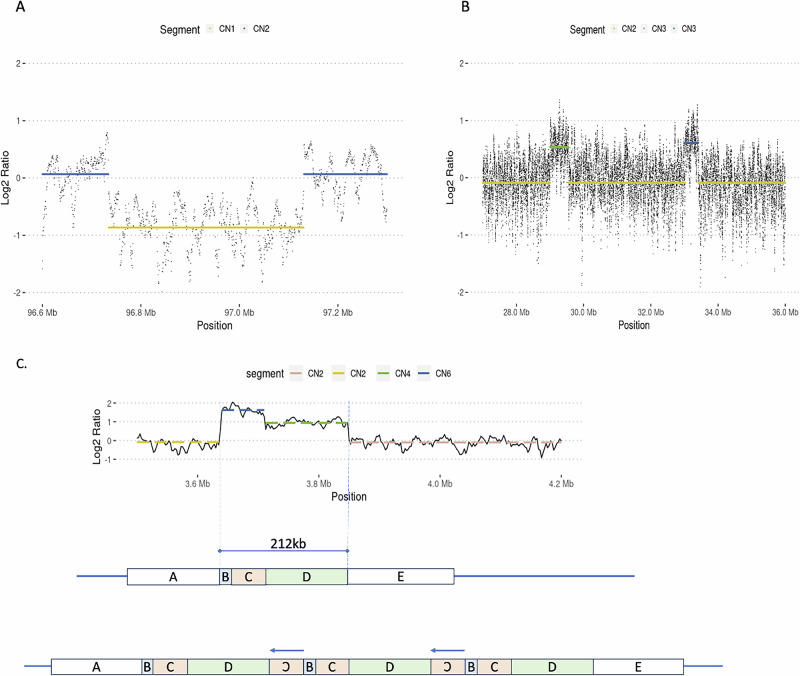
Verification of copy number changes using read depth (log2 ratio) across targeted regions. **A** Deletion on chromosome 6 (target region J). **B** Complex rearrangement with DUP-NML-DUP (target region A). **C** Top panel shows read depth across a complex rearrangement on chr16. Copy number can be inferred from the log2 read depth ratio plotted across the region. Colored lines correspond to the mean copy number within breakpoint regions. The lower panel shows the deduced structure of the complex rearrangement, using a combination of read depth and breakpoint reads (breakpoints in IGV shown in Supplementary Figures). Note that region “B” is actually smaller than depicted, and is therefore not clearly visible in the top panel log2 ratio plot.

CNV callers developed for long-read sequencing are typically not suitable for targeted sequencing approaches, such as adaptive sampling. With targeted sequencing, copy number inference can either be made in comparison to all other targeted regions, to all other samples analyzed, or to coverage on either side of the variant within the targeted region. Using only either side of the variant may lead to misinterpretation of the baseline coverage, especially in regions flanked by low copy repeats. To confirm that the results represent one deletion and not two flanking duplications, the read depth was normalized on the mean depth over the additional target regions in that sample, and the log2 ratio shows that it is a deletion and not two duplications. Two examples are shown in Fig. 3. The log2 ratio also scales well to the determination of higher copy numbers, and when combined with breakpoint information, it is possible to resolve very complex chromosomal rearrangements, as shown for a TRIP-QUINT-TRIP with inverted segments in Fig. 3C.

### Use of rejected reads for CNV calling

**Fig. 4:**
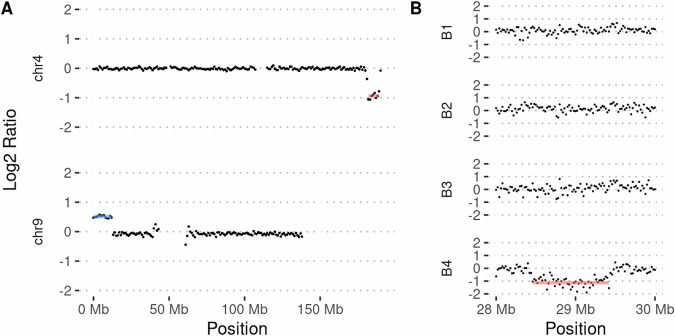
Use of rejected reads for CNV calling. **A** A copy number loss and gain on chromosomes 4 and 9, respectively, called using the rejected reads from adaptive sampling on the P2S. Target regions, common CNV regions, and centromeres/telomeres were removed from the analysis. **B** Plots of off-target read depth profiles across chromosome 16 from four samples multiplexed on a PromethION flow cell, showing a 1 Mb deletion on chr16 in barcode 4, which can clearly be distinguished as a copy number aberration using read depth data.

In addition to the high coverage of long reads over the targeted regions, the adaptive sampling gives even coverage of short ejected reads across the whole genome. The ejected reads in our study provided an overall genome coverage of 2.9–6.2x and had an N50 of 521–595 bp. To see whether these background reads could be used to scan the genome for large CNVs, we purposely excluded known CNV regions from the target reference file. The chr4:9 translocation rearrangement (regions M, N) also leads to a copy number loss of chr4 and a copy number gain on chr9, which were not targeted in the adaptive sampling. As shown in Fig. 4A, the copy number differences are clearly visible in the background coverage using ejected reads, showing near the deviation in log2 ratio that would be expected for an autosomal single copy loss (−1) and single copy gain (+0.58), respectively. To investigate a smaller region, we also removed the chr16 deletion (region O) when sequencing the patient carrying this deletion on the P2S system. Even for this smaller region (around 1MB), the deletion is detected in the background data, showing the expected one copy loss (Fig. 4B). The analysis of background reads may be useful for scanning for CNVs, e.g., after targeted gene panel sequencing using adaptive sampling, where no prior whole genome analysis for CNVs has been performed.

## Discussion

Our results indicate that adaptive sampling has great value as a rapid and flexible strategy for verification of structural variants identified by standard routine genetic diagnostic assays such as short-read WGS, exome sequencing, or clinical microarrays. The majority of the samples here had gone through WGS, but follow-up from exome or CMA results would work equally well with a proper selection of the target region. We successfully verified all the rearrangements for a variety of structural variants, including complex chromosomal rearrangements. Although the exact nucleotide resolution breakpoints could not be established for all variants, each aberration could be confirmed by a combination of read depth and breakpoint spanning reads. Adaptive sampling offers several advantages compared to targeted assays currently used for verification of SVs and complex chromosomal rearrangements, including e.g. MLPA, FISH, or Sanger sequencing. The primary advantages are that the assay is rapid and flexible, and target regions can easily be added and removed before each new sequencing run, without the need for new probes or oligos. Any region of the genome can be targeted, and multiple targets can be combined. Therefore, in addition to unique targets that may be relevant for a specific patient, reference target regions can be included for all patients, which can then be used for comparison across samples, including normalizing read depth for copy number analysis.

The long-read sequencing output is beneficial compared to probe-based assays as it provides details about breakpoints and allows resolution of rearrangement architecture, providing a more complete view of SVs and complex variants. The targeted sequencing also enables identification of SNVs and indels on the non-rearranged haplotype, making it possible to pick up additional variants in recessive disorders. While not specifically analyzed in our study, the nanopore sequencing also provides information about cytosine methylation, which can be relevant both for imprinted regions and repeat expansion targets. In addition, methylation can be used to identify skewed X-inactivation. Among our adaptive sampling target regions, we routinely include the *AR* and *RP2* genes on chromosome X (data not shown), for which methylation signatures can be used to detect skewed X-chromosome inactivation [30, 31].

With a few notable exceptions, the breakpoint junctions could readily be mapped to the GRCh38 reference by breakpoint spanning reads. For a chr16p11 deletion, the exact breakpoints within large segmental duplications could not be determined, but the CNV was confirmed by read depth analysis. In one sample, the prior analysis of the rearrangement indicated that the breakpoint resides in a sequence that is missing in GRCh38, although it had previously been narrowed down to a specific region (hg19; 95.5–96.7 in T2T-CHM13; 94.3–95.6 in hg38). Using the T2T-CHM13 reference assembly, we were able to resolve this translocation. We could determine that the chromosome 6 breakpoint is included in GRCh38, but maps to an unlocalized contig (chr6:95331948-chrUn_KI270333v1:0). With the T2T-CHM13 reference, we find the exact chr6 breakpoint, as well as the breakpoint on the short arm of chr22. This example also shows that translocations can be called without targeting both chromosomal breakpoint regions, which is important for translocations involving centromeric or subtelomeric sequences, as these are rarely sufficiently unique to include as a target in adaptive sampling. Targeting one side of a translocation breakpoint leads to lower coverage, but enables detailed mapping of breakpoints in a region of high complexity. Our results show the value of also considering the T2T-CHM13 assembly when resolving translocations, as has previously been shown in whole-genome long-read sequencing projects [21, 32]. These results highlight the importance of migrating towards a more complete reference.

In our study, we evaluated adaptive sampling with both MinION and PromethION. We chose to run four samples on a PromethION flow cell and found that the coverage per sample is approximately similar to a single sample processed on MinION. The benefits of multiplexing on PromethION are increased throughput, reduced cost per sample, and the multiplexed samples can be used as an internal reference with exactly the same experimental conditions. In our experiments, we also noted slightly better enrichment on the P2 instrument, with the integrated GPUs making more rapid decisions about ejecting fragments in the adaptive sampling. However, multiplexing on PromethION flow cells also comes with the risk of uneven sequence coverage between samples in the pool. This risk can be minimized by proper quality control, e.g., by ensuring that all samples in the pool are similar in terms of purity and fragment length distribution. Here, we only used the adaptive sampling method built into MinKNOW, but alternatives where the adaptive sampling is dynamically updated during the sequencing run to balance the barcodes are also possible [33, 34].

The average coverage in our experiments is approximately 30x for autosomal target regions using the R10 flow cells and kit 14 chemistry. Previous studies have shown that this is more than sufficient for accurate germline SNV and indel calling [16], and that even lower long-read coverage is necessary for SV calling [35]. However, for CNV calling, lower coverage may lead to challenges in distinguishing copy number, especially in low complexity regions or when there are multiple copies present. A previous study using adaptive sampling for copy number confirmation performed downsampling of target coverage and estimated that 4-5X was sufficient to call deletions and duplications in the >50–100 kb range [19]. A concern in terms of coverage is the sex chromosomes in males, as well as the detection of mosaic variation. To reach 30x autosomal coverage, we reloaded the flow cell up to two times. Reloading increases the total coverage, but also increases the run time (up to 72 hours for two re-loads). With our workflow on MinION, we performed input quality control one day, performed the library prep and sequencing over the next 2–3 days (depending on the number of re-loads), and then analyzed the data. For certain diagnostic applications, more rapid sequencing is essential [36–38], and it is then an option to run single or fewer samples on PromethION flow cells without reloading in order to increase yield. It is also possible to cut down on the time for input quality control by going straight to sequencing if the approximate DNA quality and fragment distribution are known. Further time can be saved by initializing analysis as reads are produced, especially for breakpoint validation, where a low number of breakpoint junction reads may be sufficient for variant verification. Another potential limitation for current clinical diagnostic implementation is cost. For a single MinION run, the sequencing reagent cost, in our hands, ended up at around €800. Running multiple samples on PromethION lowers the cost, but requires more coordination and runs the risk of unbalanced multiplex pools. The flow cell cost is also highly dependent on the pack size ordered. The reagent cost using nanopore for verification of structural variants is therefore more expensive than some of the existing options for diagnostic target verification assays (MLPA, qPCR, FISH), but also offers the benefits of flexibility and more rapid set-up for new targets. We note that the sequencing cost is continuously decreasing, and for some patients, a more rapid diagnosis may be worth the additional cost. We also note that all the benefits of long-reads are equally valid for whole genome sequencing with long-reads, and would argue that as cost comes down, using long-read WGS as a first-tier clinical test would become both time and cost efficient.

While adaptive sampling provides advantages in terms of flexibility, there are also limitations to consider for implementation in a diagnostic setting. One disadvantage of using adaptive sampling is that the target region size is limited. To achieve maximum sequencing yield, this should ideally be no more than 5% of the human genome (https://nanoporetech.com/document/adaptive-sampling), which could be problematic if the sample carries large copy number variations. Here, we show that large CNVs can confidently be called in the background data, indicating that it may be sufficient to only target breakpoint regions for verification of large variants, and use background coverage to validate the copy number status across the complete CNV segment. A previous study has shown the potential of using adaptive sampling as a clinical workflow for CNV confirmation [19]. Our study differs in that we include a broader range of rearrangement types that rely on read depth and breakpoint identification. In comparison, we aimed for longer reads and higher sequence coverage. Studies aiming only to confirm changes in copy number are less dependent on read length, while read length is more important when aiming to capture breakpoints. Here, we were still unable to map exact breakpoints in extended regions of low copy repeats or segmental duplications. Here we sheared the DNA to just over 10 kb, which naturally limits the ability to sequence into duplicated segments. It may be possible to resolve such regions by extracting very high molecular weight DNA, followed by sequencing of ultra-long libraries. Alternatively, target enrichment using CRISPR/Cas targeting sites in adjacent unique DNA would be a way to enrich duplicated regions. The benefit of CRISPR/Cas protocols is a significantly greater enrichment of the target over the background. However, it has limitations in that the enrichment adds additional experimental steps, and each target requires optimization. Both ultra-long libraries and CRISPR/Cas enrichment (or a combination of both) could therefore be a way to resolve breakpoints also in segmental duplication regions, which may be especially important, for e.g., identification and verification of inversions with breakpoints in long inverted repeat regions.

In this study, we used adaptive sampling for verification of germline structural variants and complex chromosomal rearrangements. The potential role for adaptive sampling in clinical diagnostics is significantly broader. The flexibility of the approach makes it excellent for gene panels that require frequent addition of new genes in rapidly evolving fields, including many areas of rare disease and cancer diagnostics. The long reads enable phasing of variants, which is relevant for recessive disorders. In addition, long reads can span across repeat expansions, making it possible to combine mutation screening and repeat sizing in the same experiment, which is highly relevant, e.g., neurodegeneration panels. The ability to also analyze cytosine methylation makes it valuable for imprinting disorders and cancer classification. We do note that the stated benefits of long-read sequencing also apply to whole-genome long-read sequencing, making it an excellent first-tier test for a wide range of diagnostic tests. As our results show, it is also possible to use the background non-target reads to identify CNVs. Such a broad range of relevant applications in combination with increasing accuracy, decreasing cost, and improved bioinformatic workflows makes adaptive sampling a very attractive and competitive solution for future genetic diagnostics.

## Supplementary information

Supplemental Material

## Data Availability

All raw sequencing data generated in this study have been submitted to the European Genome-phenome Archive (EGA) under accession number (EGAD50000001821). Sample IDs and file names are summarized in Supplementary Table S2.

## Code availability

The data in this study were processed with https://github.com/fellen31/adaptiveqc (commit hash b7877fc) and https://github.com/genomic-medicine-sweden/nallo (commit hash 08cb5ea). The underlying code and auxiliary scripts for this study are available in https://github.com/fellen31/variant_validation and 10.5281/zenodo.16678494.
