# Supplementary material for: Flexible and rapid validation of structural variation using adaptive sampling
Source: Eur J Hum Genet. 2026 Feb 23;34(5):649–57. doi: 10.1038/s41431-026-02039-4 (PMC13171962; doi:10.1038/s41431-026-02039-4)
Supplement: Supplementary file 1 — Supplemental Material [file 41431_2026_2039_MOESM1_ESM.pdf]

Supplementary Files

Supplementary Table 1.

Sample	Sex	Platform	Rearrange-ment region (Table 1)	Rearrangement	Pass bases (Gb)	Target regions included	Copy number change detected	Breakpoint junction reads identified
S1	F	MinION	A	chrXp21.1 Dup-Nml-Dup	14.3	A - G	Yes	Yes
S2	M	MinION	B	Chr14q11.2 Dup-Nml-Dup	14.6	A - L	Yes	Yes
S3	M	MinION	C	Chr16p13.3 Trip-Quint-Trip	17.0	A - G	Yes	Yes
S4	M	MinION	C	Chr16p13.3 Trip-Quint-Trip	14.1	A - O	Yes	Yes
S5	F	MinION	E	Chr4q25 Dup-Trip-Dup	15.4	A - G	Yes	Yes
S6	M	MinION	J, K	Chr6q16.1 del Chr6;22 Translocation	16.9	A - K	Yes	Yes
S7	M	MinION	L	Chr17;19 Translocation	18.3	A - K	N/A	Chr 17: Yes
S8	F	MinION	M, N, O	Unbalanced Chr4;9 Translocation Chr4 qterdel Chr9 pterdup Chr16p11.2 Deletion	15.1	A - O	Yes	Translocation: Yes Deletion: No
B1 (S3)	M	P2S	C	Chr16p13.3 Trip-Quint-Trip	8.4	A - J, L - N	Yes	Yes
B2 (S1)	F	P2S	A	chrXp21.1 Dup-Nml-Dup	9.7	A - J, L - N	Yes	Yes
B3 (S2)	M	P2S	B	Chr14q11.2 Dup-Nml-Dup	9.0	A - J, L - N	Yes	Yes
B4 (S8)	F	P2S	M, N, O	Unbalanced Chr4;9 Translocation Chr4 qterdel Chr9 pterdup Chr16p11.2 del	10.1	A - J, L - N	Yes	Translocation: Yes Deletion: No

P2S – PromethION 2 Solo, Dup – duplication, Trip – Triplication, Quint – Quintuplication, Nml – normal copy number,

Supplementary Table 2

Sample	Target Regions	File name in EGA
S1	A - G	20230717_1631_MN23293_FAX31524_8f551f59
S2	A - L	20231009_1431_MN23293_FAX56944_cbd8020b
S3	A - G	20230712_1543_MN23293_FAX32391_3d6b7754_20230710_1521_MN23293_FAX32391_27ca4d51
S4	A - O	20231023_1507_MN22993_FAX54901_9bd2d7d5_20231026_1022_MN22993_FAX54901_7d924418
S5	A - G	20230704_1535_MN23293_FAX20347_fd6867ed
S6	A - K	20230907_1404_MN22993_FAX54893_2fadf810
S7	A - K	20230919_1517_MN23293_FAX55036_c0969d5f
S8	A - O	20231026_1022_MN23293_FAX54949_91eb1ca2_20231023_1507_MN23293_FAX54949_2dfa3987
B1	A - J, L - N	p2_solo.barcode01
B2	A - J, L - N	p2_solo.barcode02
B3	A - J, L - N	p2_solo.barcode03
B4	A - J, L - N	p2_solo.barcode04

Table showing sample, target regions and the corresponding file name for files uploaded to EGA.

Supplementary Figures

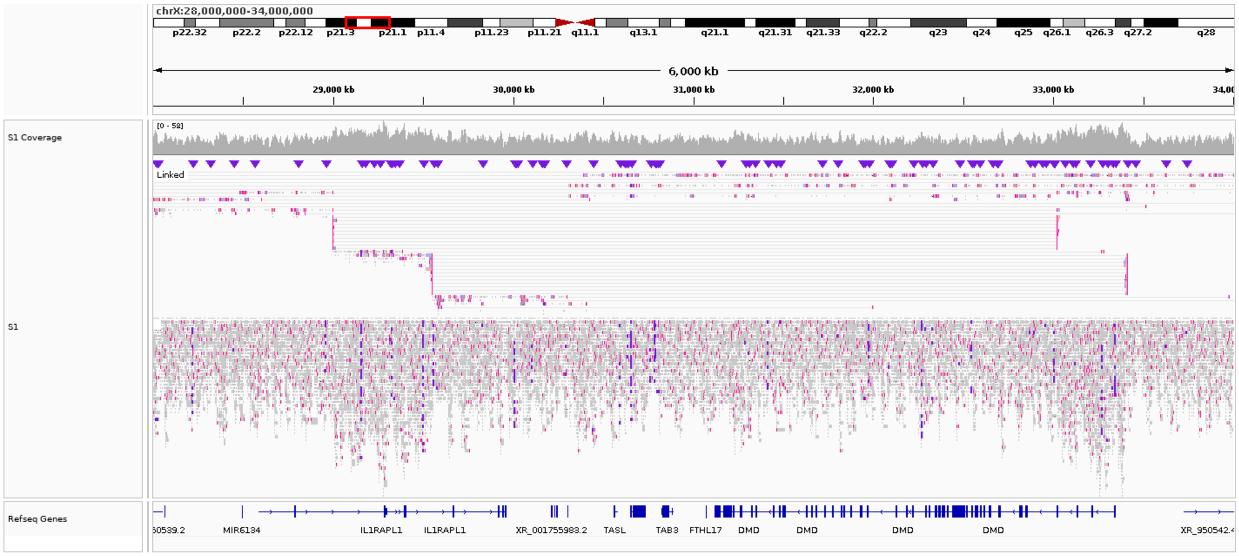

Figure S1a. Overview of highlighted rearrangement involving *IL1RAPL1* & *DMD* (Target region A) in sample S1 sequenced on MinION (same as sample B2 sequenced on P2S).

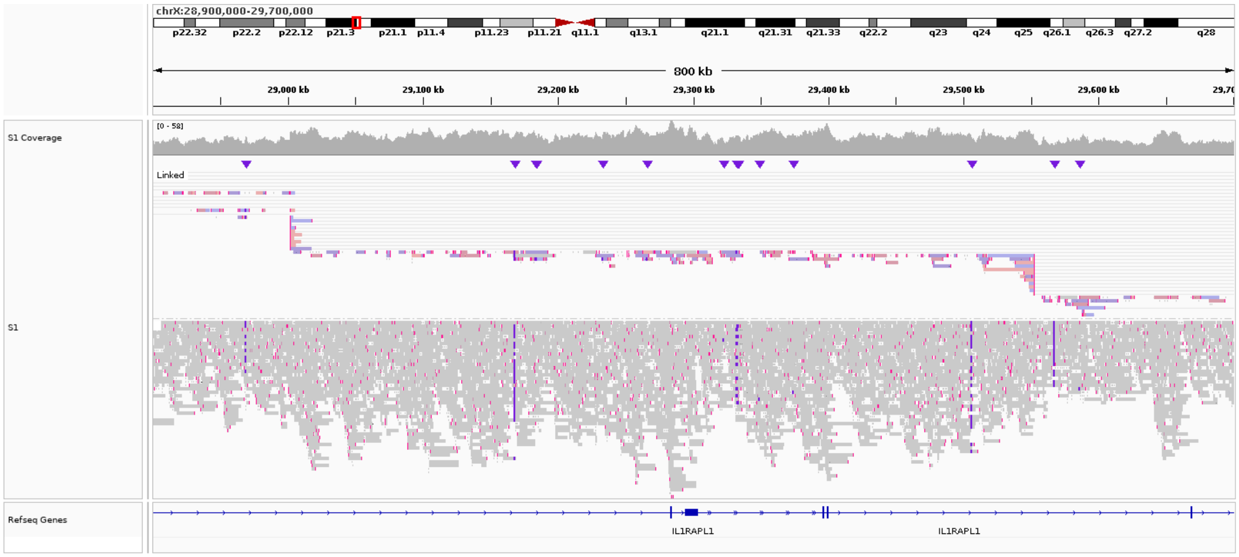

Figure S1b. Left breakpoint of highlighted rearrangement involving *IL1RAPL1* & *DMD* (Target region A) in sample S1 sequenced on MinION (same as sample B2 sequenced on P2S).

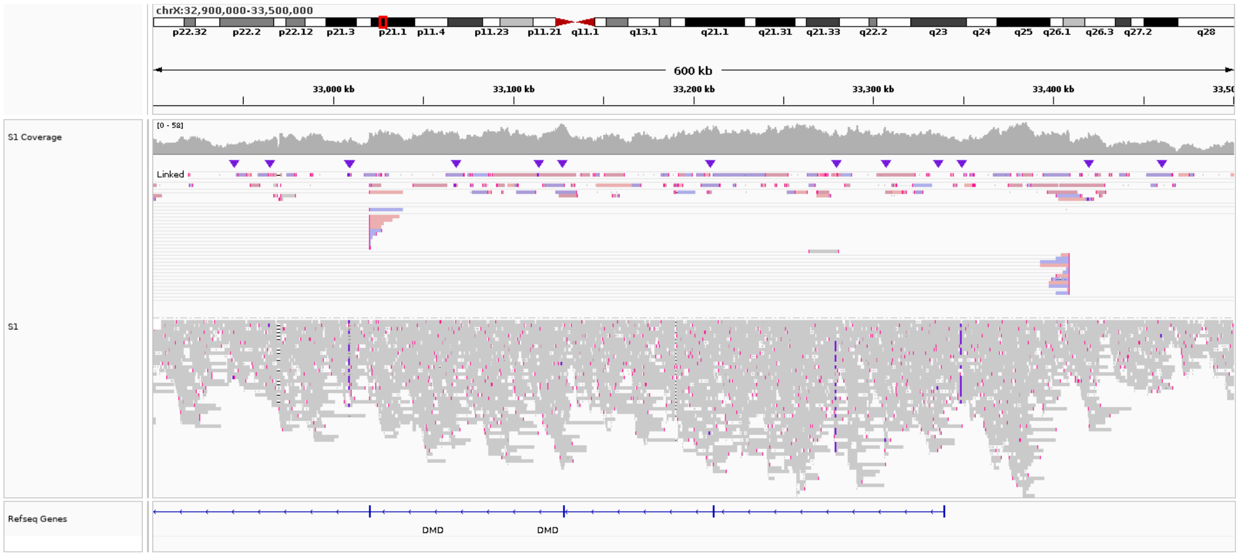

Figure S1c. Right breakpoint of highlighted rearrangement involving *IL1RAPL1* & *DMD* (Target region A) in sample S1 sequenced on MinION (same as sample B2 sequenced on P2S).

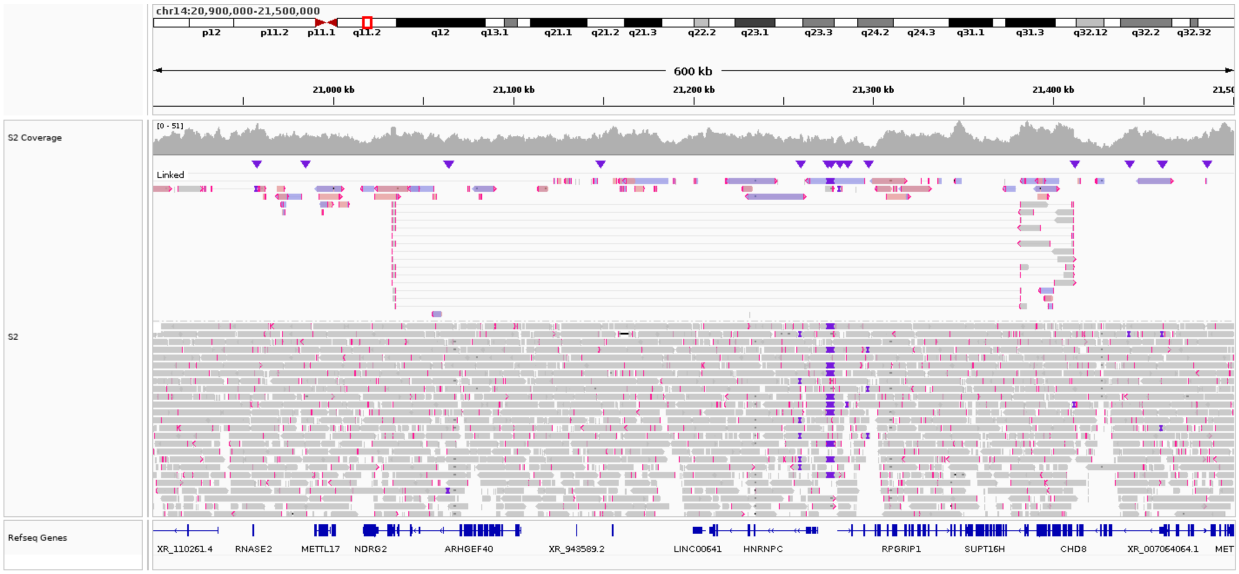

Figure S2a. Overview of highlighted rearrangement involving *CHD8* (Target region B) in sample S2 sequenced on MinION (same as sample B3 sequenced on P2S).

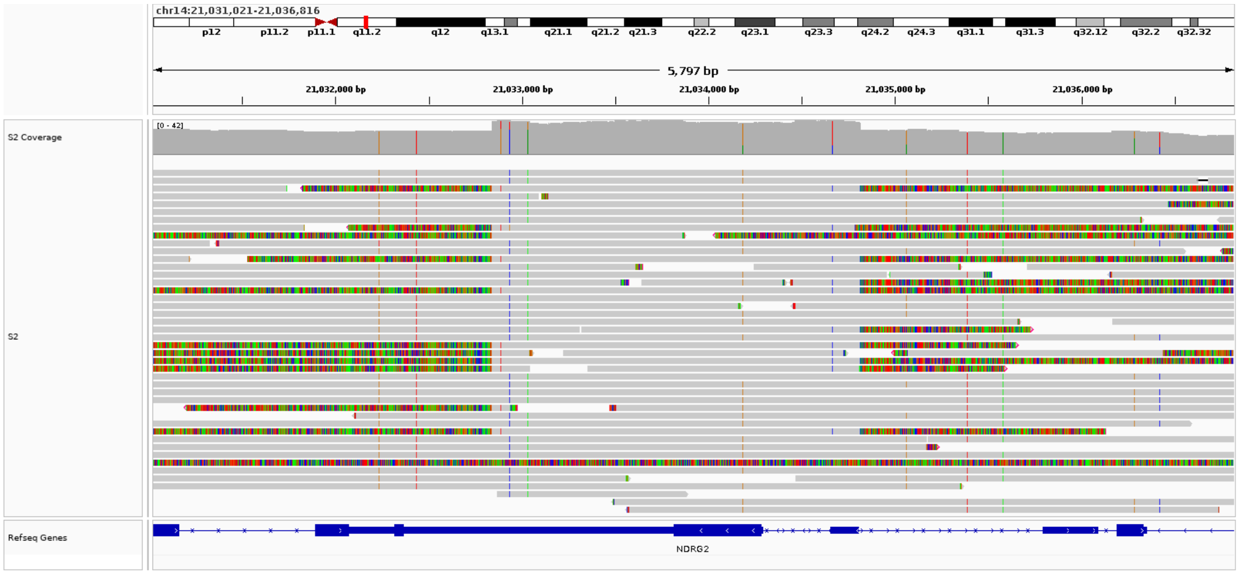

Figure S2b. Left breakpoint of highlighted rearrangement involving *CHD8* (Target region B) in sample S2 sequenced on MinION (same as sample B3 sequenced on P2S).

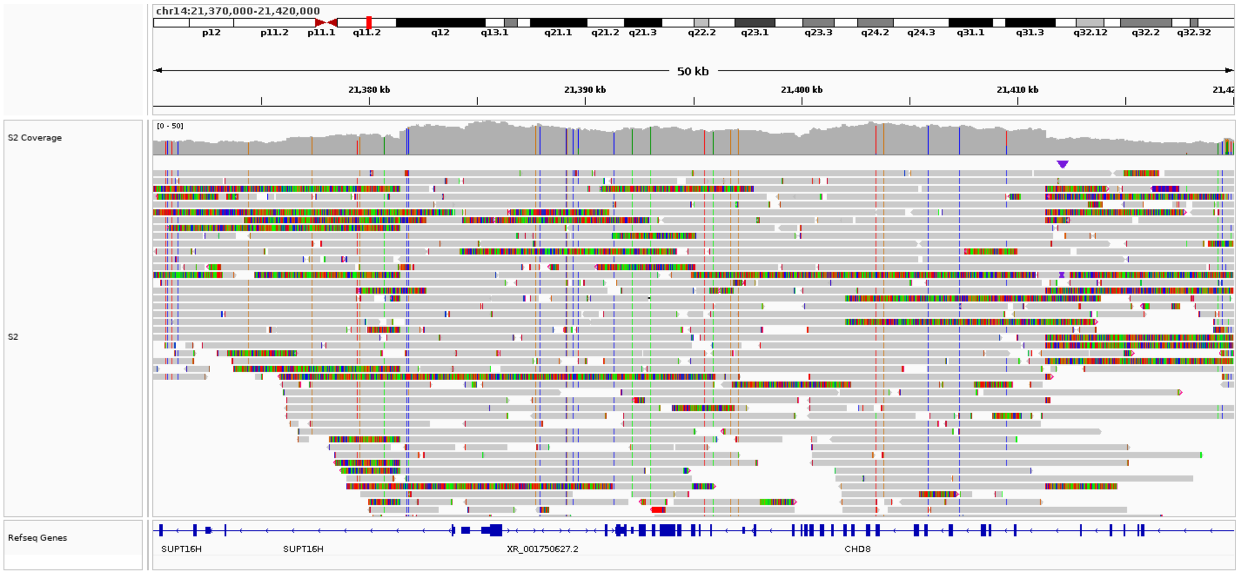

Figure S2c. Right breakpoint of highlighted rearrangement involving *CHD8* (Target region B) in sample S2 sequenced on MinION (same as sample B3 sequenced on P2S).

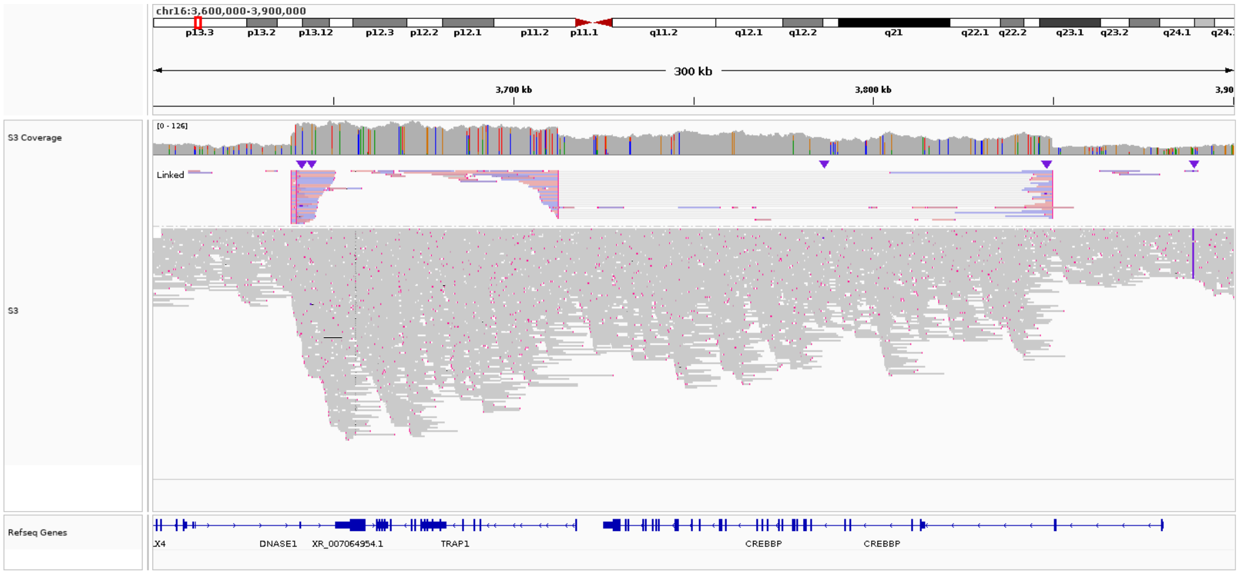

Figure S3a. Overview of highlighted rearrangement involving *CREBBP* (Target region C) in sample S3 (Father) sequenced on MinION (same as sample B3 sequenced on P2S).

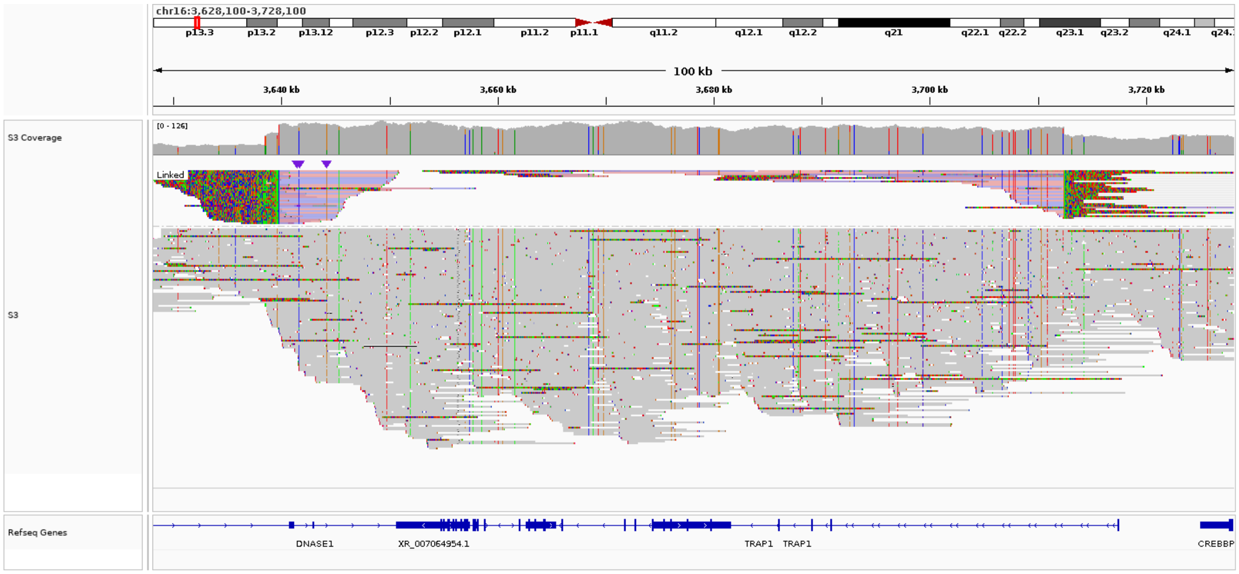

Figure S3b. Left breakpoint of highlighted rearrangement involving *CREBBP* (Target region C) in sample S3 (Father) sequenced on MinION (same as sample B3 sequenced on P2S).

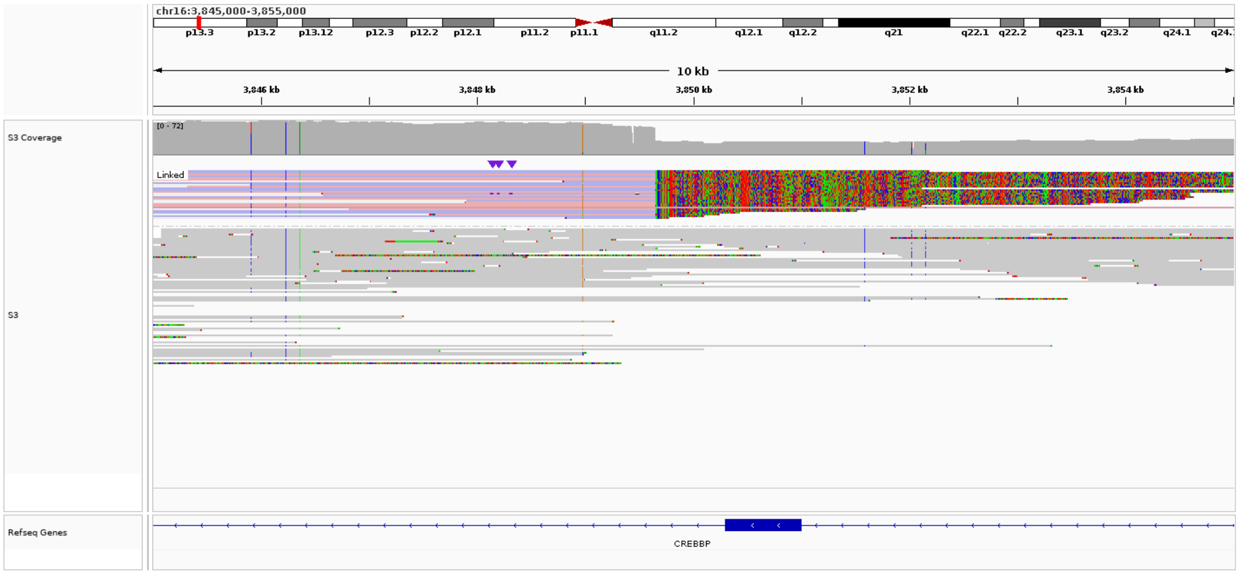

Figure S3c. Right breakpoint of highlighted rearrangement involving *CREBBP* (Target region C) in sample S3 (Father) sequenced on MinION (same as sample B3 sequenced on P2S).

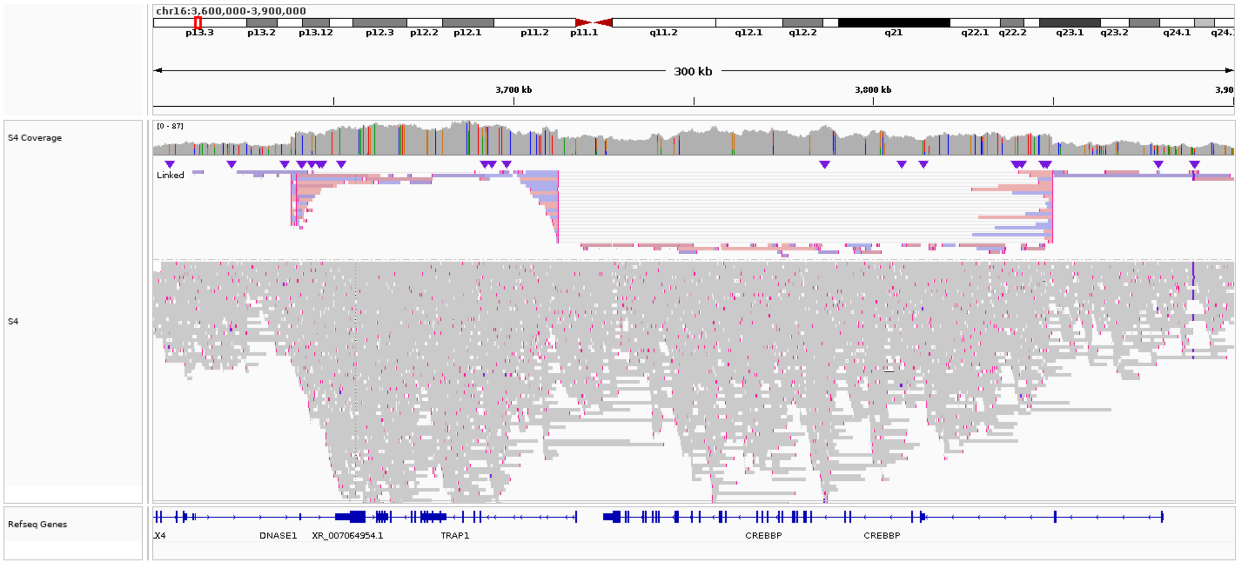

Figure S4a. Overview of highlighted rearrangement involving *CREBBP* (Target region C) in sample S4 (Proband) sequenced on MinION.

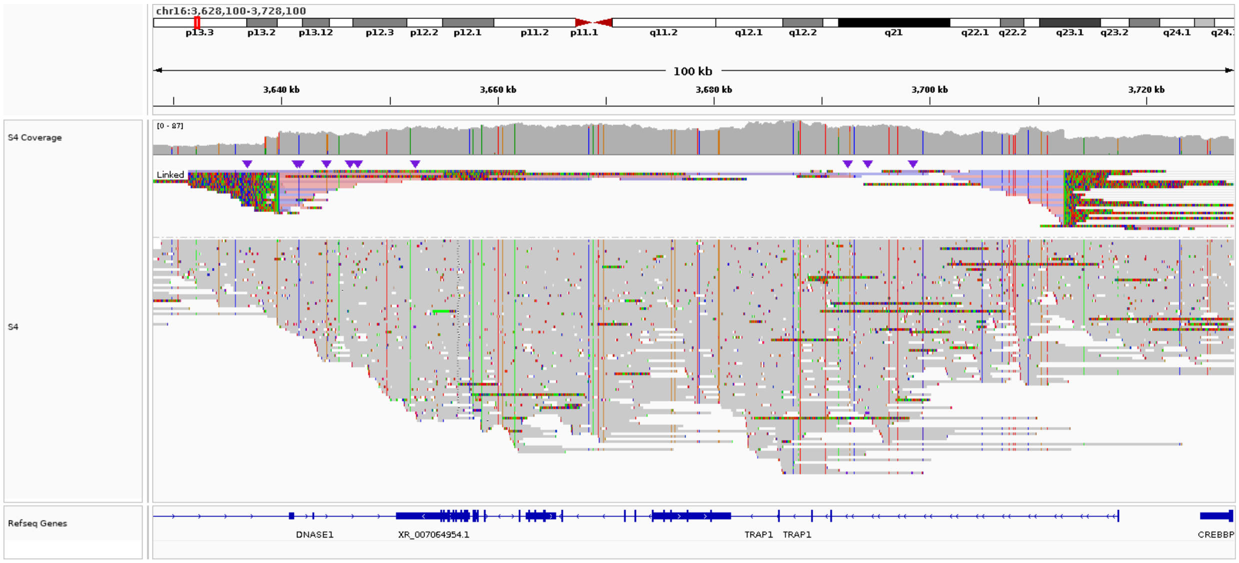

Figure S4b. Left breakpoint of highlighted rearrangement involving *CREBBP* (Target region C) in sample S4 (Proband) sequenced on MinION.

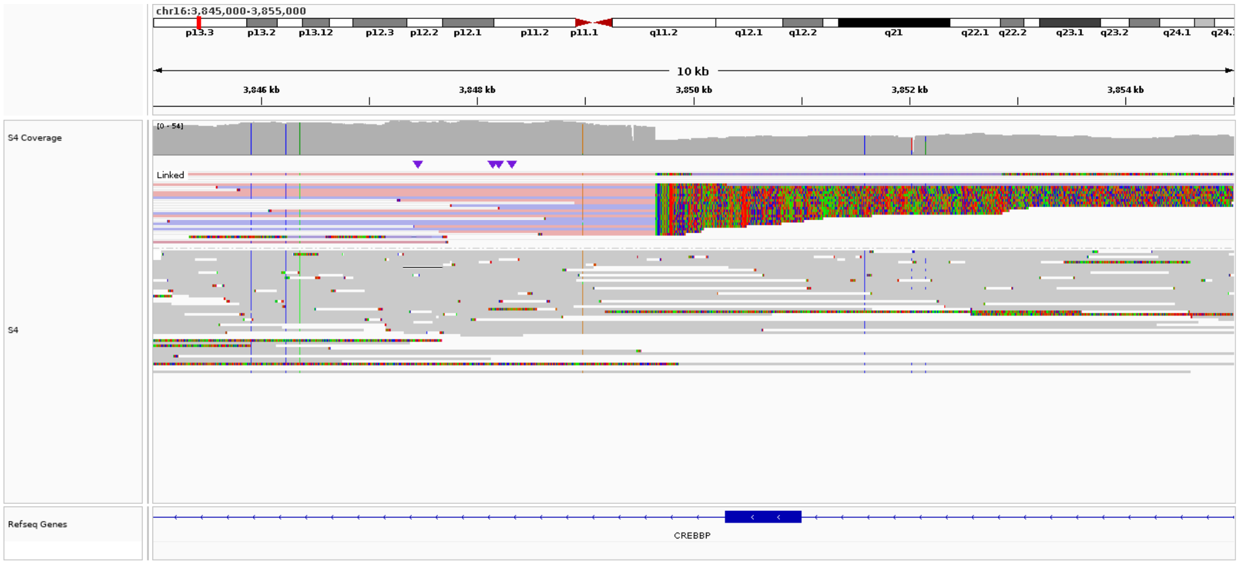

Figure S4c. Right breakpoint of highlighted rearrangement involving *CREBBP* (Target region C) in sample S4 (Proband) sequenced on MinION.

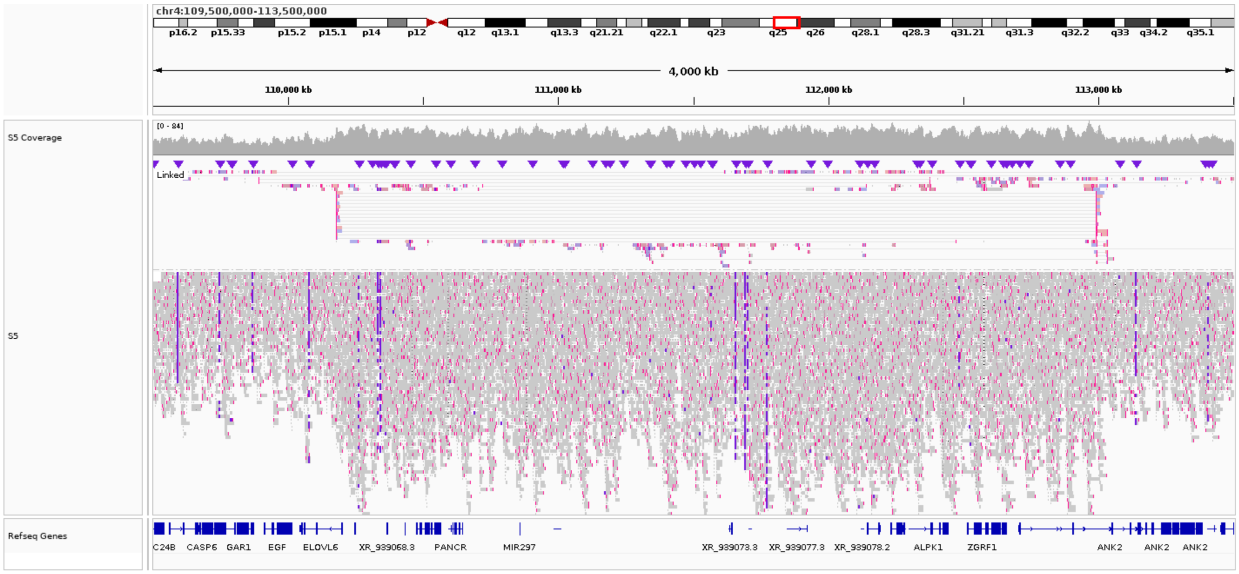

Figure S5a. Overview of highlighted rearrangement involving *ANK2* (Target region E) in sample S5 sequenced on MinION.

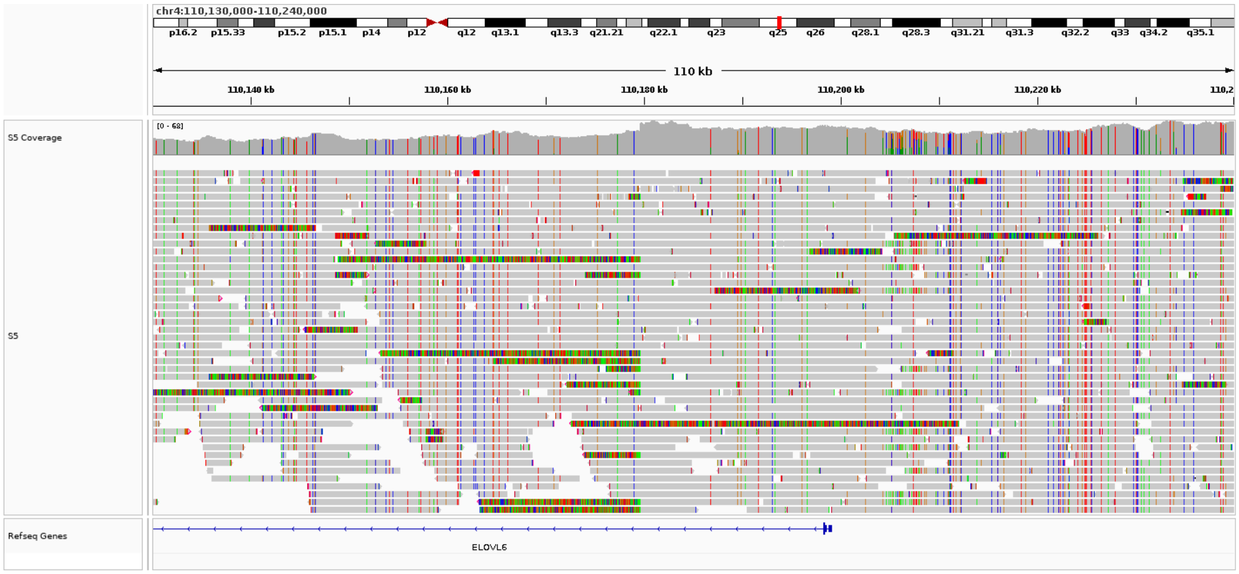

Figure S5b. Left breakpoint of highlighted rearrangement involving *ANK2* (Target region E) in sample S5 sequenced on MinION.

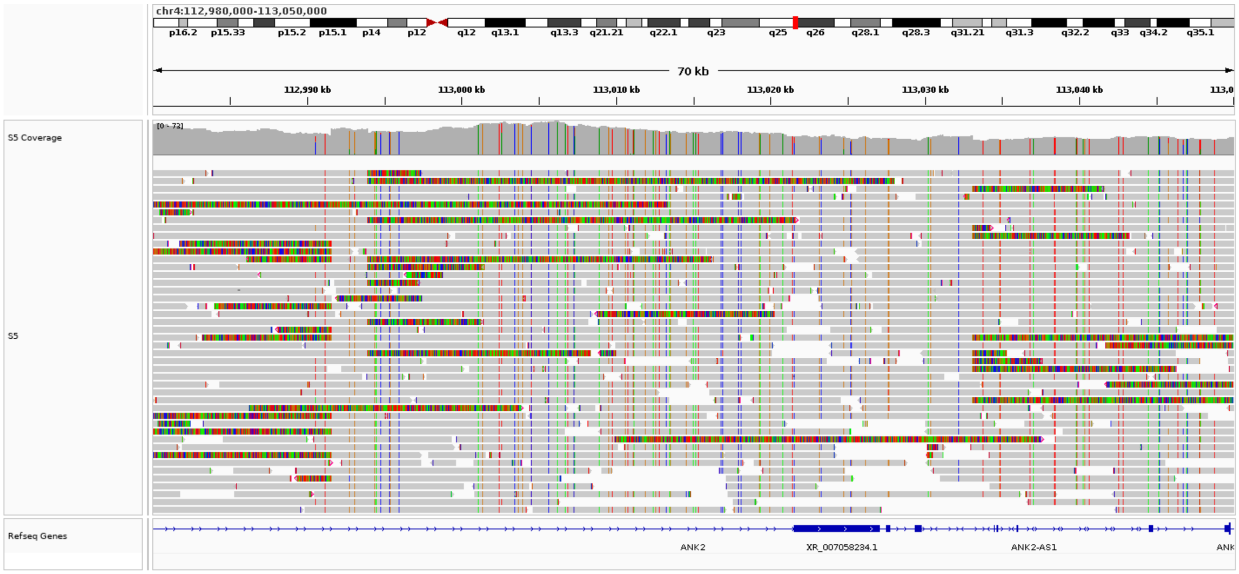

Figure S5c. Right breakpoints of highlighted rearrangement involving *ANK2* (Target region E) in sample S5 sequenced on MinION.

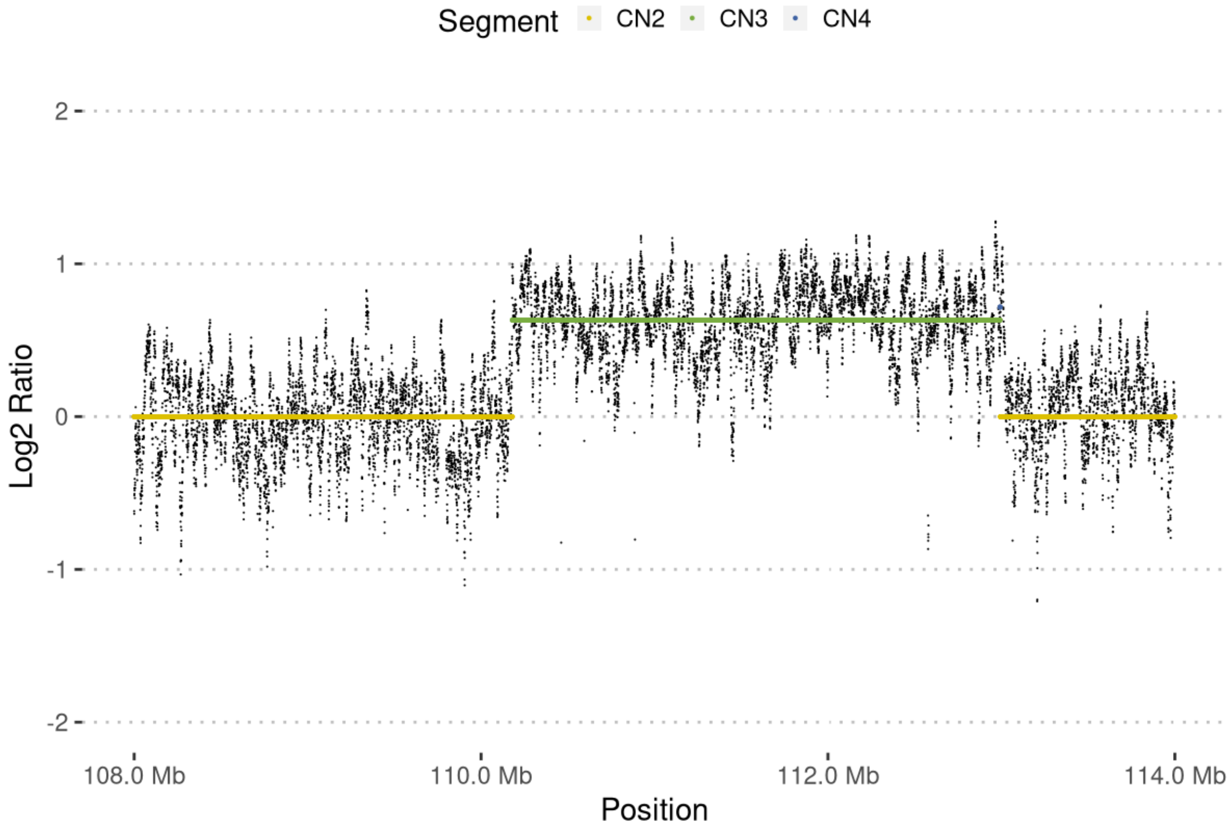

Figure S5d. Coverage plot of highlighted rearrangement involving *ANK2* (Target region E) in sample S5 sequenced on MinION.

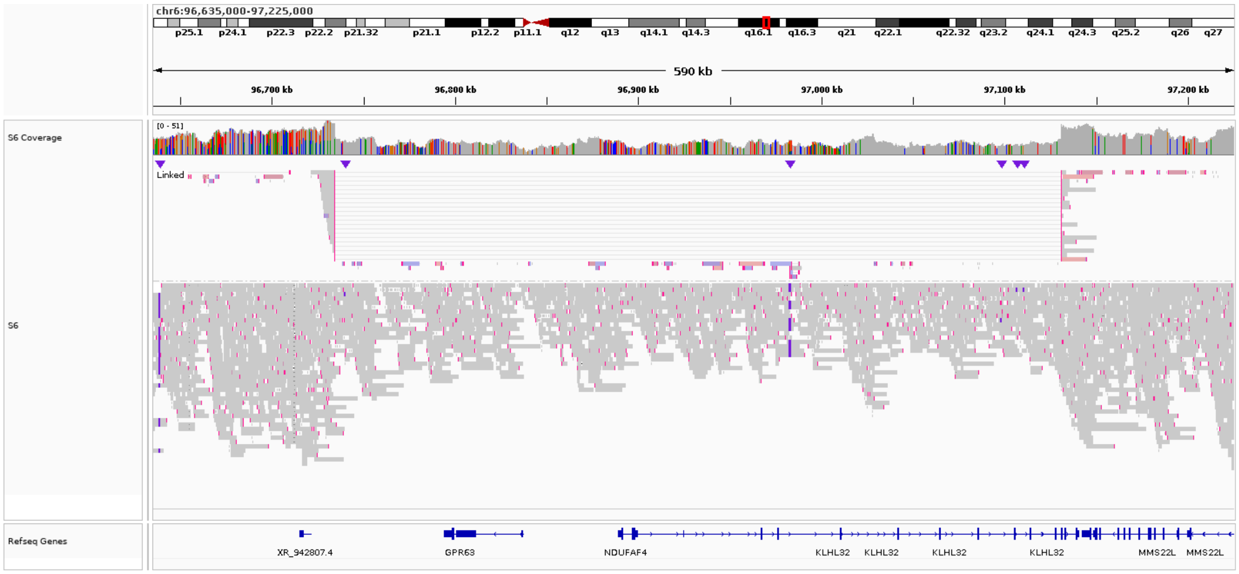

Figure S6a. Overview of highlighted rearrangement involving a deletion (Target region J) in sample S6 sequenced on MinION.

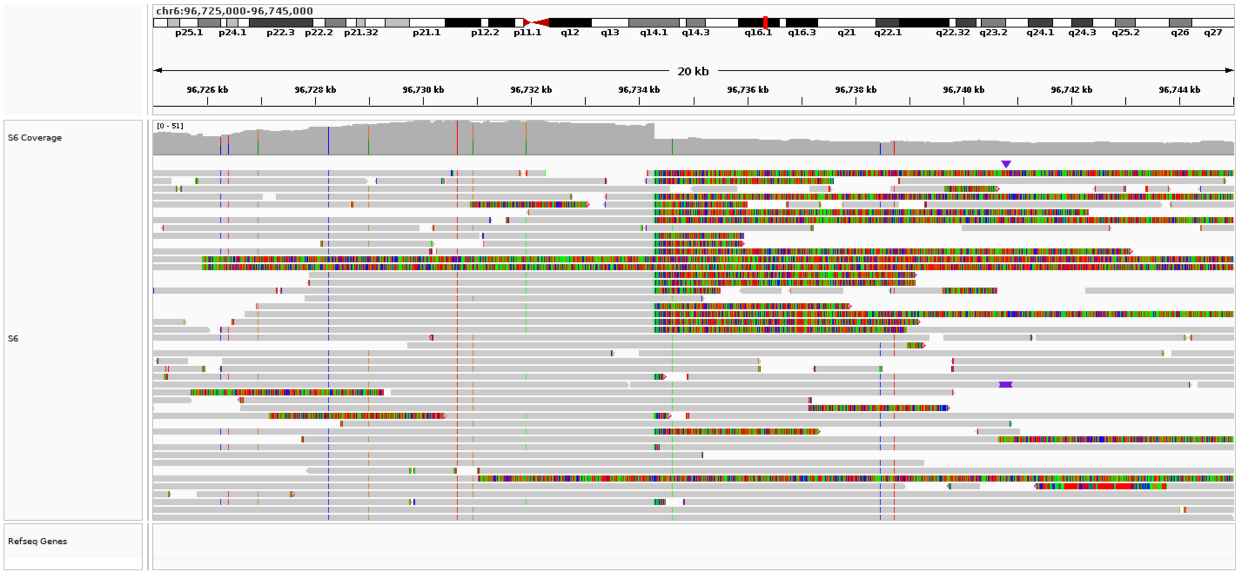

Figure S6b. Left breakpoint of highlighted rearrangement involving a deletion (Target region J) in sample S6 sequenced on MinION.

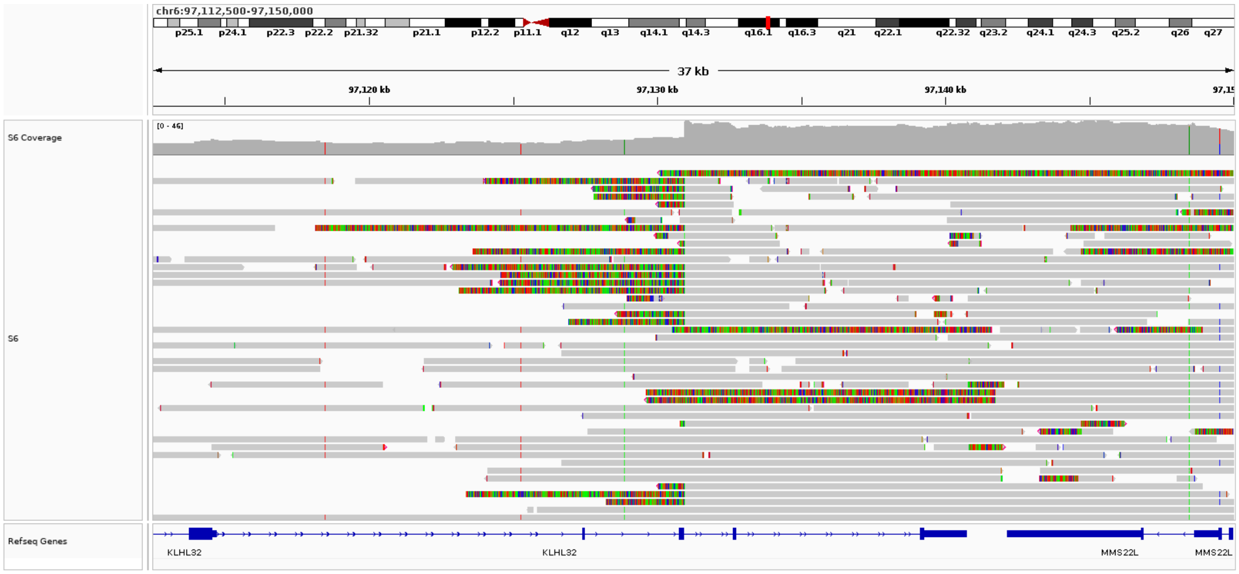

Figure S6c. Right breakpoint of highlighted rearrangement involving a deletion (Target region J) in sample S6 sequenced on MinION.

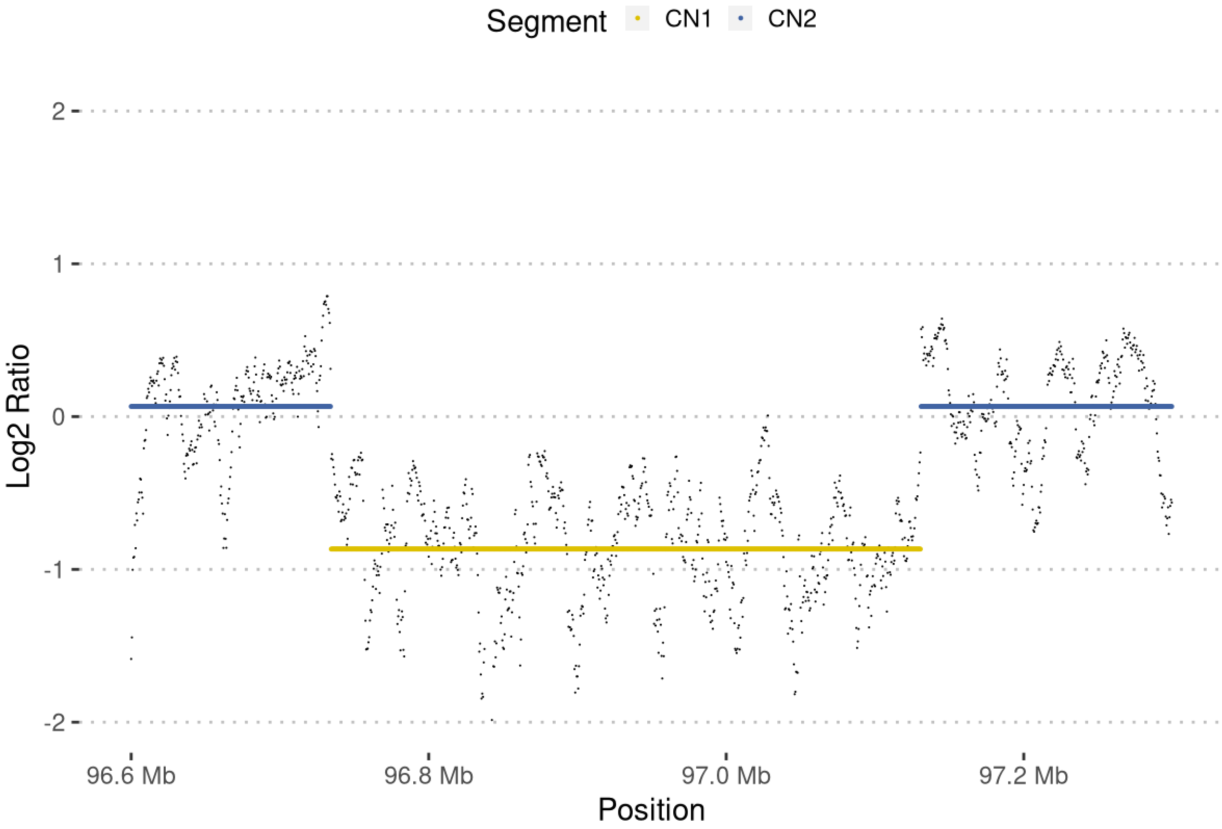

Figure S6d. Coverage plot of highlighted rearrangement involving a deletion (Target region J) in sample S6 sequenced on MinION.

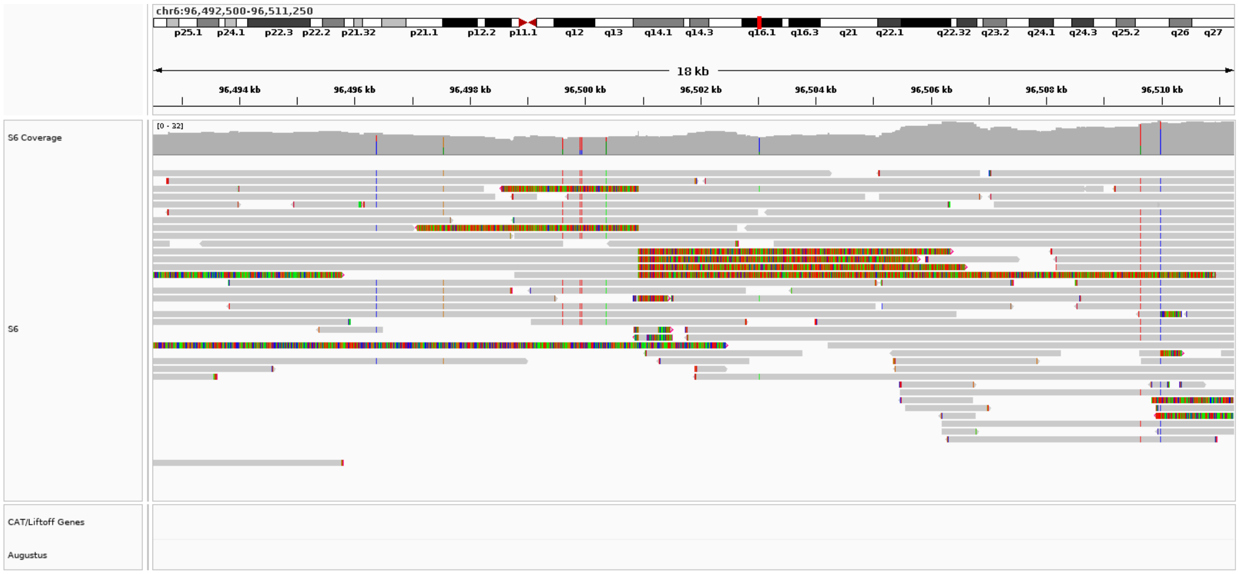

Figure S6e. Overview of highlighted rearrangement involving a translocation (Target region K) in sample S6 sequenced on MinION.

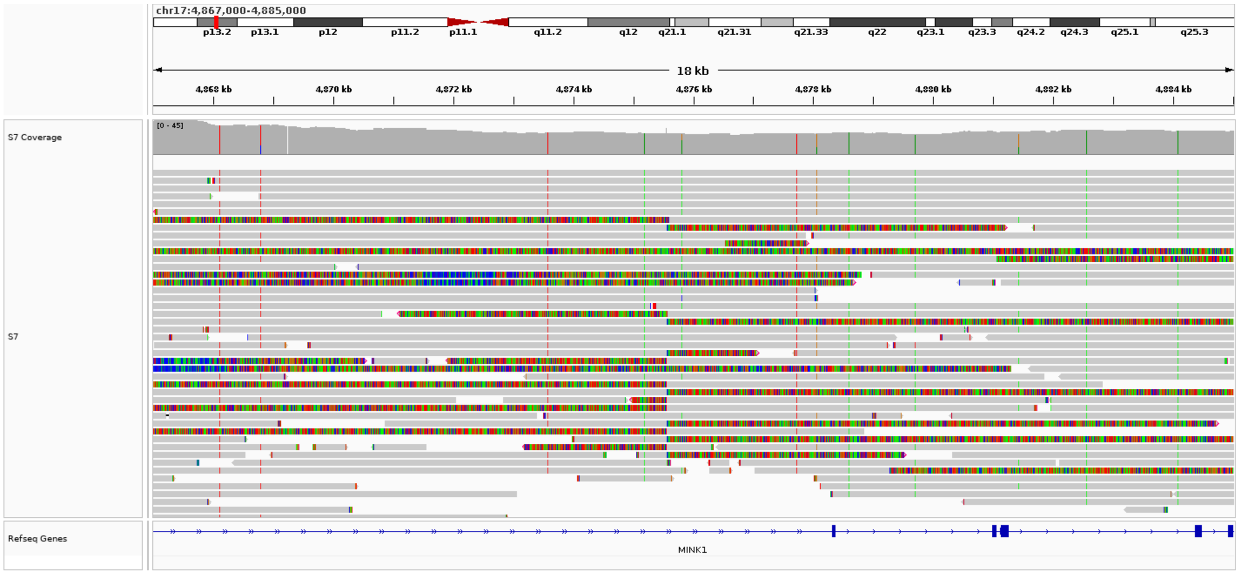

Figure S7a. Overview of highlighted rearrangement involving a translocation (Target region L) in sample S7 sequenced on MinION.

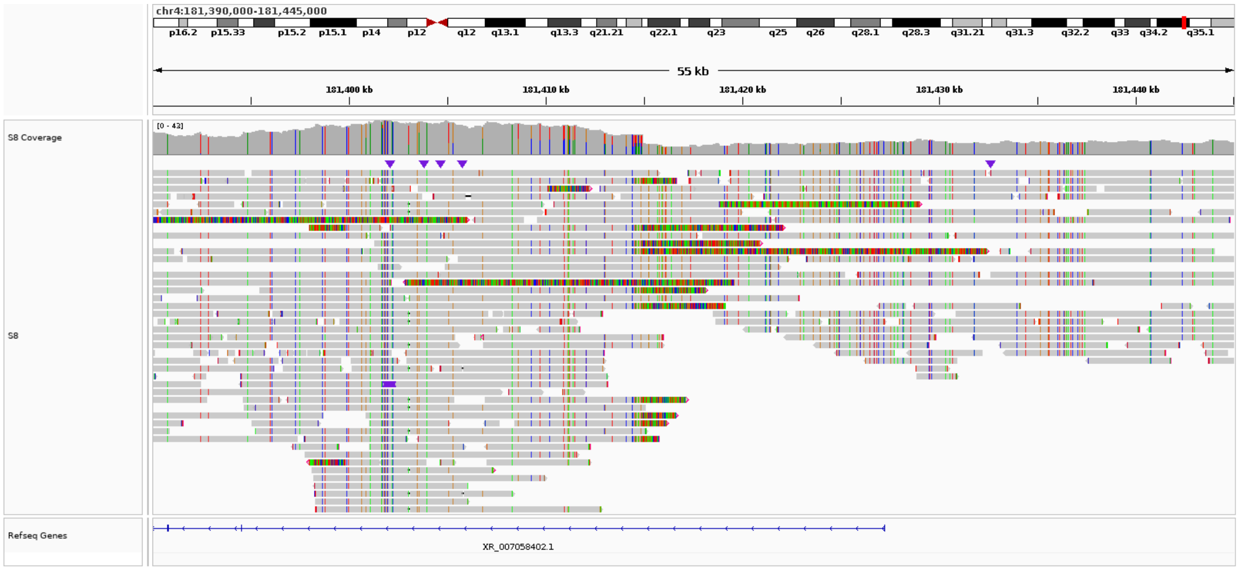

Figure S8a. Overview of highlighted rearrangement involving a translocation (Target region M) in sample S8 (same as B4) sequenced on MinION.

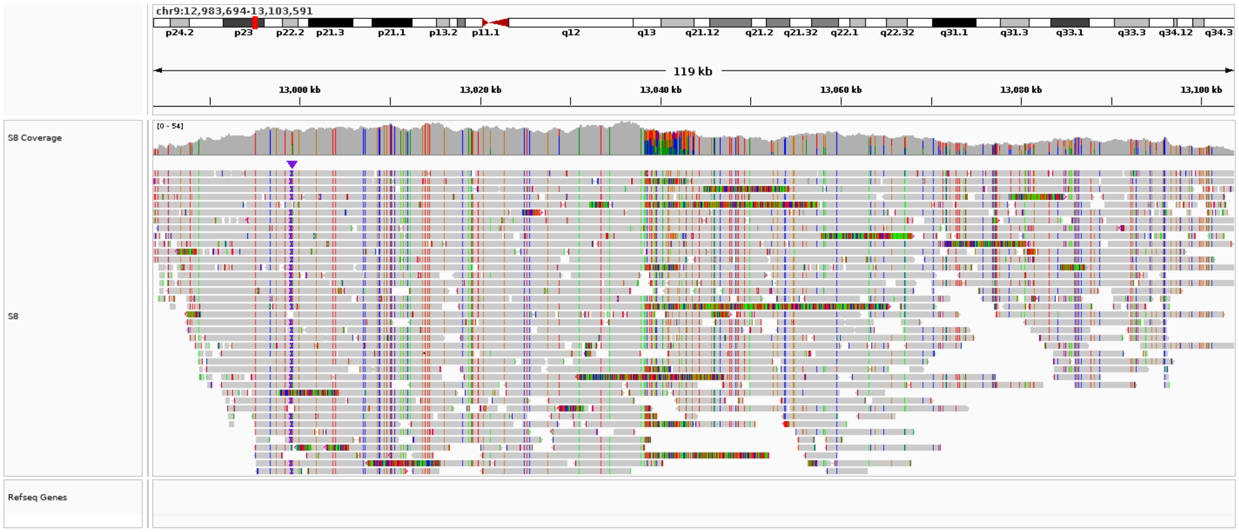

Figure S8b. Overview of highlighted rearrangement involving a translocation (Target region N) in sample S8 (same as B4) sequenced on MinION.

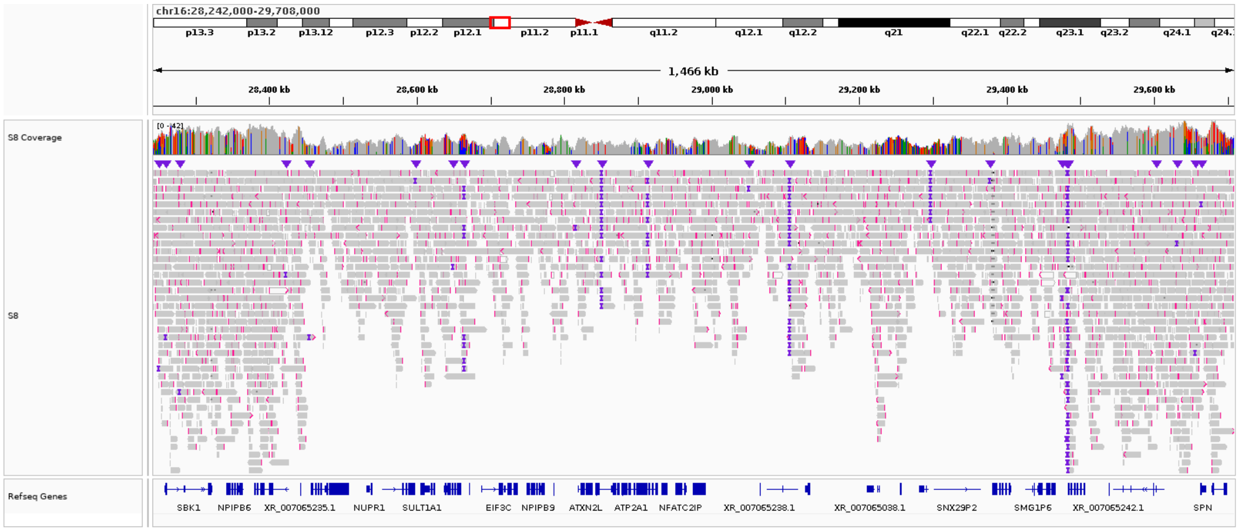

Figure S8c. Overview of highlighted rearrangement involving a deletion (Target region O) in sample S8 (same as B4) sequenced on MinION.

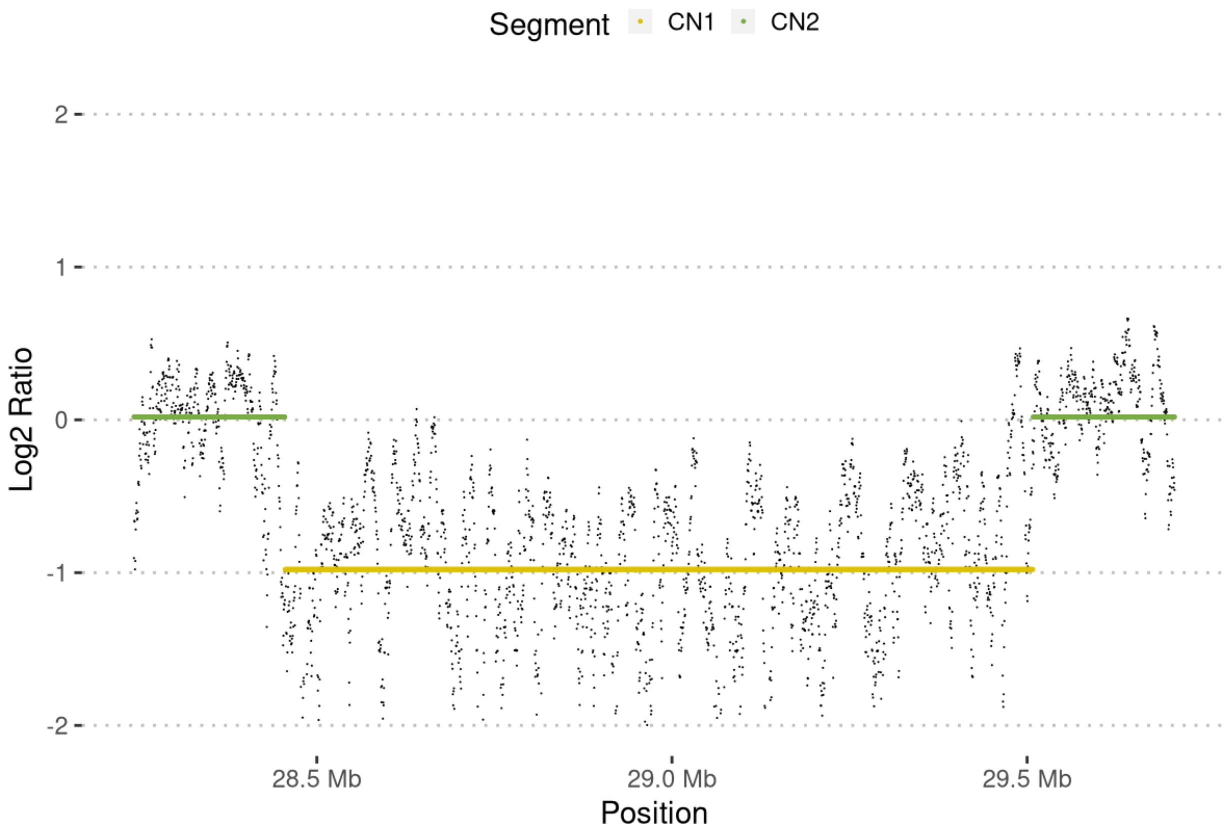

Figure S8d. Coverage plot of highlighted rearrangement involving a deletion (Target region O) in sample S8 (same as B4) sequenced on MinION.
